# Label-free quantitative SWATH-MS proteomic analysis of adult myocardial slices in vitro after biomimetic electromechanical stimulation

**DOI:** 10.1038/s41598-022-20494-z

**Published:** 2022-10-03

**Authors:** M. A. Zabielska-Kaczorowska, A. E. Bogucka, K. Macur, P. Czaplewska, S. A. Watson, F. Perbellini, C. M. Terracciano, R. T. Smolenski

**Affiliations:** 1 Department of Biochemistry, Medical University of Gdansk, Gdansk, Poland; 2 Department of Physiology, Medical University of Gdansk, Gdansk, Poland; 3 Intercollegiate Faculty of Biotechnology, University of Gdansk and Medical University of Gdansk, Gdansk, Poland; 4 Institute of Biochemistry, Medical Faculty, Justus Liebig University of Giessen, Giessen, Germany; 5 National Heart & Lung Institute, Imperial College London, London, UK; 6 Hannover Medical School, Institute of Molecular and Translational Therapeutic Strategies, Hannover, Germany

**Keywords:** Proteins, Proteomics, Cardiovascular biology

## Abstract

A special in vitro model maintained with ultrathin cardiac slices with a preserved architecture, multi-cellularity, and physiology of the heart tissue was used. In our experiments, we performed label-free quantitative SWATH-MS proteomic analysis of the adult myocardial slices in vitro after biomimetic electromechanical stimulation. Rat myocardial slices were stretched to sarcomere lengths (SL) within the physiological range of 1.8–2.2 μm. Electromechanically stimulated slices were compared with slices cultured without electromechanical stimulation (unloaded and nonstimulated-TW) on a liquid–air interface and with fresh myocardial slices (0 h-C). Quantitative (relative) proteomic analyses were performed using a label-free SWATH-MS technique on a high-resolution microLC-MS/MS TripleTOF 5600+ system (SCIEX). The acquired MS/MS spectra from the DDA LC–MS/MS analyses of the rat heart samples were searched against the UniProt Rattus norvegicus database (version of 15.05.2018) using the Paragon algorithm incorporated into ProteinPilot 4.5 (SCIEX) software. The highest number of differential proteins was observed in the TW group—121 when compared to the C group. In the 1.8 and 2.2 groups, 79 and 52 proteins present at a significantly different concentration from the control samples were found, respectively. A substantial fraction of these proteins were common for two or more comparisons, resulting in a list of 169 significant proteins for at least one of the comparisons. This study found the most prominent changes in the proteomic pattern related to mitochondrial respiration, energy metabolism, and muscle contraction in the slices that were stretched and fresh myocardial slices cultured without electromechanical stimulation.

## Introduction

In vitro models are widely developed, well-established, and simple approaches in many biological studies. After many years of experiments in the artificial environment, there was a need to create something more, a bridge between in vivo experiments and cellular models. Many novel achievements in heart tissue preparation and in vitro culture methodology are attracting academic, commercial, and industrial interest. For instance, living myocardial slices (LMS) are an interesting platform to study, which gives a fresh look at cardiac physiology. LMS are extremely thin slices of living ventricular tissue^1^. The thickness of 100–400 μm remains physiologically active and preserves a specific architecture and multicellularity of the heart. Because of the special properties, it is possible to treat slices of the myocardium with a mechanical and electrical load, drugs, or biological compounds^2,3^. It was discovered that using electromechanical stimulation and physiological preload improves the maintenance of myocardial function and structure^4^. The technique of the preparation is very well optimized and results in 97% of viable cardiomyocytes with the proper conduction, contractility, and Ca^2+^ handling^5^. It was found by Watson et al. that a preload equivalent to sarcomere length (SL) of 2.2 μm is optimal for the maintenance of the structural, functional, and transcriptional properties of the myocardium in the 24 h culture. Rat LMS had significant upregulation of gene sets associated with transcription and translation, as well as cardiac structure and function. Moreover, gene sets associated with extracellular matrix organization, inflammation, and cytokine signaling were significantly downregulated. As a result, the proper Ca^2+^ handling, the optimal maintenance of maximum contractility, and t-tubule structure were observed. Any changes in viability or energy status were noticed.

Over the last few decades, liquid chromatography coupled to tandem mass spectrometry (LC–MS/MS) has become the methodology of choice for the highly sensitive characterization of proteins and proteomes in a variety of biological samples. In 2012, Gillet et al. described a special relative quantification technique—Sequential Windowed Acquisition of All Theoretical Fragment Ion Spectra (SWATH-MS)^6,7^. SWATH-MS is an appropriate technique for quantitative label-free analysis of complex protein and peptide mixtures. In the previous studies, we used a SWATH-MS method to allow label-free quantification of virtually any number of proteins in one analysis^8^.

## Objectives

Proteomic patterns in the heart slices under biomimetic electromechanical stimulation have never been studied before. Therefore, we performed the study with freshly prepared rat myocardial slices and subjected them to different electromechanical stimulation protocols for 24 h. Electro stimulated slices which were stretched to sarcomere lengths within the physiological range of 1.8 μm (1.8) and 2.2 μm (2.2) and slices cultured without electromechanical stimulation on a liquid–air interface (unloaded and nonstimulated-TW) were compared with fresh myocardial slices (0 h-C). Quantitative (relative) proteomic analyses of heart samples were performed using a label-free SWATH-MS technique on a high-resolution microLC-MS/MS TripleTOF 5600+ system (SCIEX). In this study, we searched for a number of protein biomarkers between all the tested groups compared to fresh myocardial slices cultured without electromechanical stimulation. We focused on the changes in the proteins related to energy metabolism, mitochondrial respiration, and muscle contraction.

## Methods

### Experiments on animals and tissue preparation

All animal experiments complied with institutional and national regulations in the UK. The study was reported in accordance with ARRIVE guidelines and was approved by the ethics committee of Imperial College London. The use of living cardiac tissue was approved by Imperial College London. The procedures were described in our previous publication and were performed under license by the UK Home Office, following the United Kingdom Animals (Scientific Procedures) Act 1986. Animals were sacrificed following guidelines established by the European Directive on the protection of animals used for scientific purposes (2010/63/EU). Male Sprague Dawley rats, with a body mass 300–500 g, were sedated with isoflurane (4% isoflurane and 4 L/min O_2_), then sacrificed using cervical dislocation and dissection of the carotid arteries. Rat myocardial slices were electromechanically stimulated and stretched within the physiological range to SL of 1.8 and 2.2 μm. Stimulated (1.8, 2.2) and unloaded/non-stimulated (TW) heart slices were compared with fresh rat heart slices (C). Preparation of the rat hearts was fully described in the publication of Watson et al. (2019)^5^.

### Materials for proteomic analysis

Acetonitrile and water were purchased at Merck (Darmstadt, Germany). Formic acid was from Thermo Fisher Scientific (Waltham, MA, USA). All the reagents used in the LC–MS/MS experiments were LC–MS grade unless otherwise specified. The proteolytic enzymes (trypsin and LysC) used to digest the proteins were purchased from Promega (Germany).

### Sample preparation for proteomic analysis

Fresh-frozen heart slices were prepared for MS analysis using pressure cycling technology (PCT) in a PBI NEP2320 Barocycler (Pressure BioSciences Inc., South Easton, Easton, MA, USA)^9^. Briefly, proteins were extracted in the barocycler using a solution containing 8 M urea and 100 mM NH4HCO3 for 60 cycles of 30,000 psi for 50 s followed by 14.7 psi for 10 s per cycle at 33 °C. Disulfide bridges were reduced by incubation with 100 mM dithiothreitol at 35 °C for 30 min, and cysteine residues were alkylated by 200 mM iodoacetamide in a 30-min incubation at room temperature in the dark. Proteins were digested in the barocycler at 35 °C, first by LysC in a 1:50 enzyme–substrate ratio and next, by trypsin in a 1:20 enzyme–substrate ratio for 45 and 90 pressure cycles, respectively of 20,000 psi for 50 s followed by 14.7 psi for 10 s per cycle. Proteolytic peptides resulting from digestion were desalted on the C18 resin in the STAGE Tips procedure^10^. The desalted vacuum-evaporated samples were dissolved in 33 μL of 50% acetonitrile/0.1% formic acid. From each particular sample, 3 μL of the solution was taken to create four pool samples: C, TW, 1.8 and 2.2. The samples were transferred to HPLC vials with inserts and subjected to LC–MS/MS analyses.

## LC–MS/MS analyses

### Chromatography (LC)

Chromatographic separation of the digested samples was performed with the use of the Ekspert microLC 200 system (Eksigent, Redwood City, CA, USA) on the Eksigent ChromXP C18CL (3 μm, 120 A, 150 × 0.5 mm) column. The CTC Pal Autosampler (CTC Analytics AG, Zwinger, Switzerland) was employed for the injection of 5 μL of the sample onto the column. Mobile phases were composed of 0.1% (v/v) formic acid in water (A) and 0.1% formic acid in acetonitrile (B). Peptide mixtures were separated in the column using the following gradient program: (i) 0–2 min–2% phase B; (ii) 2–50 min–2–30% phase B; (iii) 50–54 min–30–98% phase B; (iv) 54–56 min–98% phase B; and (v) 56.1–60 min–2% phase B at the flow rate of 5 µl/min.

### Tandem mass spectrometry (MS/MS)

The microLC system was in-line coupled to a hybrid TripleTOF 5600+ mass spectrometer equipped with a DuoSpray Ion Source (SCIEX, Framingham, MA) set at 300 °C. The samples were analyzed in a positive ion mode (ISVF 5.5 kV). The APCI positive calibration solution (SCIEX, Framingham, MA, USA) was delivered through the APCI probe by the Calibrant Delivery System for external mass calibration. The microLC-MS/MS system was controlled by SCIEX Analyst TF 1.7 software.

#### LC–MS/MS analyses in DDA mode (data-dependent acquisition)

LC–MS/MS analyses in DDA mode were conducted for pool rat heart samples: C, TW, 1.8, and 2.2. The TOF MS survey scan was performed using an m/z range of 100–1400 with 50 ms accumulation time, and the 10 most intensive precursor ions, of + 2 to + 5 charge, were selected for fragmentation using collision-induced dissociation (CID). Product ion scans were performed in high sensitivity mode, over a 100–2000 m/z range, with a 40 ms accumulation time and rolling collision energy. Precursor ions were excluded from repeated selection for fragmentation for 5 s after two occurrences. The data was acquired for 50 min. The cycle time was equal to 1.11 s. Qualitative DDA analyses, necessary for the creation of spectral ion libraries for SWATH-MS quantification, were acquired in two replicates (separate digestions of the same sample), and for each replicate, three technical replicates were acquired (separate LC–MS/MS analyses in DDA mode).

#### SWATH-mode LC–MS/MS analysis

For each individual rat heart sample from the C, TW, 1.8, and 2.2 groups, LC–MS/MS analyses in SWATH-MS mode (data-independent acquisition—DIA) were performed. The SWATH-MS1 survey scan was acquired in high sensitivity mode, over a 100–1400 m/z range, with a 50 ms accumulation time. Product ion scans were performed over a 400–1000 m/z range divided into 25 windows of 25 Da width, wherein sequentially in each window all the precursor ions were fragmented in a looped mode. The accumulation time was equal to 40 ms and the collision energy was individually calculated for each window for + 2 to + 5-charged ions with a spread of 2. The data was acquired for 50 min. The cycle time was equal to 1.11 s. The SWATH quantitative analyses were performed in two replicates (separate digestions of the same sample), and for each replicate, three technical replicates were acquired (separate LC–MS/MS analyses in SWATH mode).

## Data processing

### Database search

The acquired MS/MS spectra from the DDA LC–MS/MS analyses of the rat heart samples were searched against the UniProt Rattus norvegicus database (version of 15.05.2018) using the Paragon algorithm incorporated into ProteinPilot 4.5 (SCIEX) software. The following database search parameters were applied: iodoacetamide cysteine alkylation; enzyme: trypsin; special factors: urea denaturation; ID focus: biological modifications; search effort through ID; detected protein threshold [Conf] > 10%; automatic false discovery rate (FDR) analysis. A protein identification analysis was performed for all the types of pool rat heart protein extracts: C, TW, 1.8 and 2.2, by a combined search of the MS/MS spectra from the. wiff files of three technical replicates. The resulting group file constituted the spectral ion library used in SWATH-MS quantification. Protein identifications were filtered at a level of 1% FDR.

### SWATH-MS data processing and normalization using the Total Protein Approach

Data from SWATH-MS experiments were processed against the MS/MS spectral ion library obtained through database search in the same manner as previously described^11^. Briefly, the group file from ProteinPilot was loaded into SWATH 2.0 in PeakView 2.2 software (SCIEX), for automatic spectral ion library creation with modified peptides allowed and shared peptides excluded. Applied SWATH processing parameters were as follows: maximum 6 peptides per protein; maximum 6 transitions per peptide; [Conf] 99; FDR < 1%; XIC extraction window: 10 min; XIC width: 75 ppm. Retention time calibration was performed based on 9–11 peptides evenly distributed along the chromatographic retention time. Manual selection of peptides and transitions for quantification depending on their quality was performed for each spectral library. Next, after automatic data processing, peptides and transitions that did not meet the applied criteria were removed. SWATH intensities for all quantified proteins were then exported to Excel and absolute protein concentrations were calculated using the Total Protein Approach (TPA) in a DIA-TPA methodology applied before^12,13^.

## Data analysis

Protein concentrations [pmol/mg] were imported into the Perseus 1.6.14 software^14^. Technical replicates were averaged by the median, and two replicates by the arithmetic mean. Principal Component Analysis (PCA) was performed and fold changes of all test groups (1.8, 2.2, and TW) to the control group (C) were calculated. Concentration values were then log2-transformed, normalized by z-score, and subjected to a t-test comparison between each test group and the control group. Statistically significant changes were regarded as the *p* value < 0.05. Heatmaps were created using average sample concentrations in the ClustVis tool^15^. Functional enrichment analysis was conducted on the DAVID 6.8 resource^16^. Interaction networks were constructed in Cytoscape 3.8.2 software using data from the STRING database^17,18^.

## Results

We were able to analyze 399 proteins using SWATH-MS quantification out of 677 proteins identified in the spectral library (Table S1–2). The absolute protein concentrations calculated by the Total Protein Approach from the normalized SWATH-MS intensities for each MS measurement spanned more than 4 orders of magnitude (Fig. S1). The proteins present at the highest and lowest analyzed concentration in the analyzed material were myosin light chain 3 (control group median: 1704.87 pmol/mg) and cytoplasmic dynein 1 heavy chain 1 (control group median: 0.19 pmol/mg). To take a general look at the differences between the study groups, we conducted a principal component analysis (PCA) of the samples (Fig. S2). The control and TW samples clustered more closely in their groups, which were completely separated from each other. On the other hand, the 1.8 and 2.2 samples were moderately scattered along the main component axis (45.6% of variation). However, the 2.2 samples tended to cluster more closely with the control group, hinting at potential similarities between those groups and differences with the TW group.

**Figure 1:**
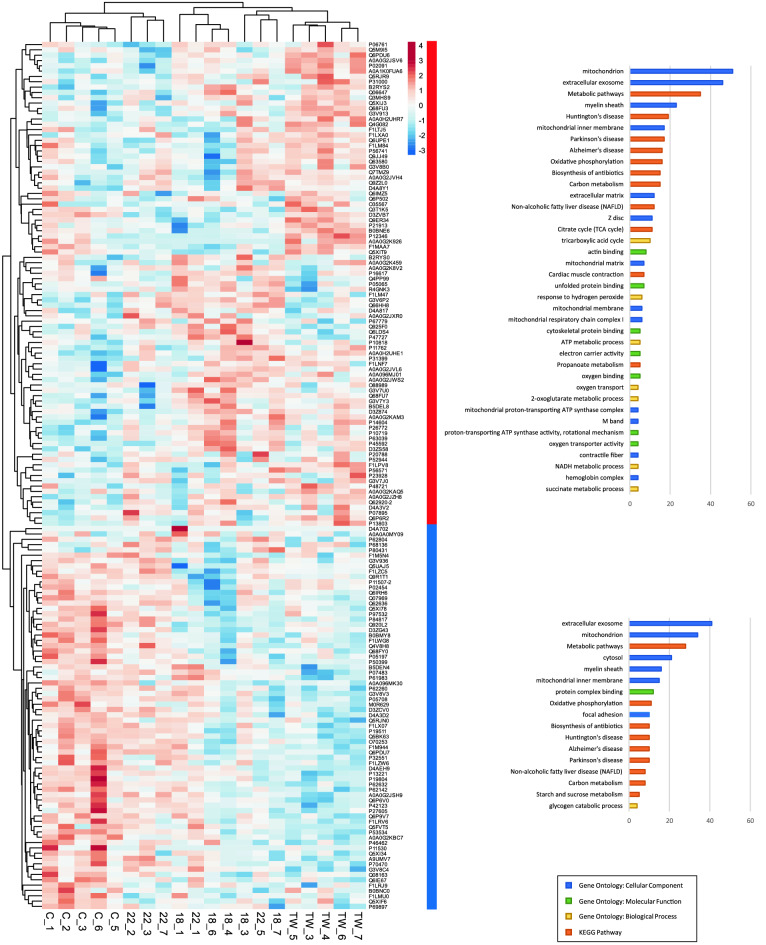
Heatmap of median protein concentrations in samples for proteins with a statistically significant concentration change in at least one of the considered comparisons to the control group (*p* value < 0.05 in a t-test). Protein lists from two main row clusters (designated by red and blue colors) were subjected to functional analysis, and the results are presented on charts, showing numbers of proteins assigned to significantly enriched terms with a Bonferroni correction adjusted *p* value < 0.05.

**Figure 2:**
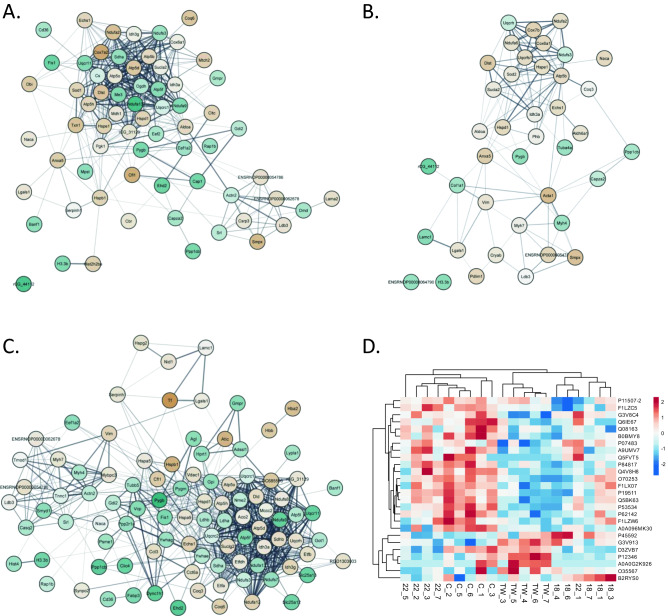
(**A**–**C**) Interaction networks of proteins with statistically significant changes in concentration in each comparison: (**A**) 1.8 for to control group, (**B**) 2.2 for to control group, and (**C**) TW for to control group. Node color designates the direction and extent of the concentration fold change: gold—FC > 1, blue—FC < 1. Node edges designate association with mitochondrion (red) or muscle structure/contraction (green). (**D**) Heatmap of median protein concentrations in samples for proteins with a statistically significant twofold or higher concentration change in at least one of the considered comparisons to the control group (*p* value < 0.05 in a t-test).

We conducted separate t-tests between each group (TW, 1.8, 2.2) and the control group (C) and considered each concentration difference with a *p* value lower than 0.05 as significant. The detailed results are listed in Table S3. We observed the highest number of differential proteins in the TW group—121. In the 1.8 and 2.2 groups, we found 79 and 52 proteins present at a significantly different concentration from the control samples, respectively. A substantial fraction of these proteins were common for two or more comparisons (Fig. S3), resulting in a list of 169 proteins for at least one of the comparisons. We constructed a heatmap for these 169 proteins using median concentrations of each sample and found two main column clusters: one containing all C samples with three 2.2 samples and the other consisting of the rest of the examined samples (Fig. 1). Rows were also divided into two main clusters: the upper cluster containing proteins with higher concentrations in the samples of the control group cluster (C samples and three 2.2 samples) and the bottom cluster, including proteins with lower concentrations outside of the control group cluster, designated by red and blue rectangles in Fig. 1, respectively. The results of the functional analysis for row clusters presented in Fig. 1 show similarities between the functions of proteins in both clusters, e.g., association with mitochondrion, extracellular exosome, metabolic pathways, oxidative phosphorylation, or carbon metabolism.

We constructed interaction networks from proteins statistically significant in each comparison (Fig. 2A–C). Proteins in each network tended to assemble into two interaction clusters to a different degree based on the total number of proteins in a given network, resulting in the most relaxed network for the 2.2 to C comparison (Fig. 2B). The tighter cluster was closely associated with the mitochondrion, while the other, more relaxed cluster consisted of proteins related to muscle contraction (marked by red and green edges in Fig. 2A–C, respectively).

To discern which protein concentration changes were the most meaningful for the cells, we restricted the list of all significantly different proteins to proteins with at least twofold changes in concentration and presented the results in Table S2. This resulted in 27 proteins: 19 for the TW to C group comparison, 12 for the 1.8 to C group comparison, and a single protein—osteoglycin—for the 2.2 to C group comparison. We constructed a similar heatmap to the one present in Fig. 1 based on the obtained list of 27 proteins and achieved an even clearer division into two-column clusters, with one of them consisting of all control and four 2.2 samples, as expected. The majority of these proteins were present at higher concentrations in the "control group" cluster, except for 5 proteins. The protein with the most increased concentration in any of the test groups was serotransferrin, with a 2.75-fold change in the TW group, whereas the most decreased concentration was noted for alpha-1-inhibitor III in the case of the 1.8 group (0.25-fold change).

## Discussion

Electromechanical stimulation of the heart slices has been previously shown to affect many metabolic pathways and gene expressions^2–4^. A broad range of techniques, such as cell mechanics, immunohistochemistry, electrophysiology, biochemistry, and calcium imaging, were employed in the studies of cardiomyocytes and heart slices^19,20^. Therefore, for the first time, the proteomic analysis of the electromechanically stimulated myocardial slices was done. Several changes in the proteome of the heart slices subjected to the different in vitro protocols were demonstrated. In our study, the 2.2 group was the most similar to the C samples. The opposite TW group was the most different from the C heart slices (Figs. 1, 2, Table S2) which is in line with the results of Watson et al. in 2019. There are statistically significant changes between the tested groups and the C group overlap or fulfil similar functions to some degree. C and TW are clearly separated, and 2.2 clusters closer to C. Heart slices C and 2.2 were clustered together. Proteins were clustered into two sets: upper marked in red—proteins present at a higher concentration in the TW group in respect to control, and bottom marked in blue—proteins present at a lower concentration in the TW group in respect to control; general functional analysis shown for each set. Most of them are associated with the mitochondrion, some with muscle structure and contraction. Analysis of the C and 2.2 groups demonstrated an increased proteomic content of mitochondrial enzymes responsible for fatty acid uptake and oxidation, then involved in the Krebs cycle as well as the proteins of the respiratory chain, suggesting increased mitochondrial abundance. In our previous study using TOM20 staining and confocal microscopy, no changes in the mitochondrial structure, function, or density were observed^5^. Interestingly, the ATP concentration measured using high-performance liquid chromatography was maintained in 1.8 and 2.2 slices when TW slices showed reduced ATP concentration. The mechanical load of myocardial slices bigger than 2.0 μm preserved the ATP: ADP ratio when compared to the C group. Reduced ATP concentration, structural (t-tubular structure) and functional (contractility and Ca^2+^ handling) remodeling in the unloaded slices may explain at least some of the proteomic changes we observed. We found that the proteome of C and 2.2 slices showed an increase in the few mitochondrial ATP synthases like Atp5b, Atp5d, and Atp5h when compared to 1.8 and TW slices. ATP synthase is an enzyme that produces ATP from ADP in the presence of a proton gradient across the membrane, which is generated by electron transport complexes of the respiratory chain23. It was shown earlier, that down-regulation of the ATP synthase is an ATP sparing mechanism in ischemia and other pathological states^21^.

Interestingly, our proteomic data matched the data from RNA sequencing obtained earlier9. In the TW group, there was a significant downregulation of the gene sets associated with energy production, such as oxidative phosphorylation, cellular respiration, and the TCA cycle. Many gene sets, like genes associated with cardiac structure and function or muscle contraction, such as extracellular matrix, t-tubules, A and I-bands, intercalated disc, Ca^2+^ handling, cardiomyocyte conduction and repolarization of the cardiomyocyte, were downregulated. We had similar observations with PCA—general differences between samples. A heatmap of median concentrations of each sample for proteins with a statistically significant change in any comparison shows even more similarities between the 2.2 and C groups. Figure 2A,B,C presenting the interaction networks for proteins statistically significant for each comparison of the test group to control, showed the smallest number of proteins different from C in the 2.2 group. This is also shown in the Venn plot in Fig. S3. Moreover, the highest number of different proteins for TW compared to C was found. In groups 1.8 and TW we noticed a downregulation of the major constituent of microtubules, Tub4a (tubulin alpha-4A chain) protein. Tub4a mutation diminishes microtubule repolymerization capability^22^. Moreover, the protein Ppp1cb (serine/threonine-protein phosphatase PP1-beta catalytic subunit)-protein phosphatase (PP1) was found downregulated in comparison to C slices. This protein is essential for cell division, muscle contractility, and protein synthesis. It participates in the regulation of glycogen metabolism, and is involved in the regulation of long-term synaptic plasticity and ionic conductances. Moreover, a few isoforms of NADH dehydrogenase, which function in the transfer of electrons from NADH to the respiratory chain, were down-regulated when compared to the C group^22–24^. Figure 2 shows many similar proteins present in the interaction networks, most of them associated with mitochondrion (red), and some with muscle structure/contraction (green). Many enzymes that are involved in a respiratory chain like NADH dehydrogenases (Ndufa6, Ndufa3, Ndufa2, Ndufa13), cytochrome c oxidase subunit 7B (Cox7b) or cytochrome b-c1 complex subunit (Uqcrfs1) were up in the proteomic analysis when 2.2 was compared to the C group. For example, inhibiting complex I NDUFA4L2 was found to protect against mitochondrial dysfunction and ischemia/reperfusion‐induced cardiomyocyte apoptosis^25^. Interestingly, mitochondrial superoxide dismutase (Sod2), which destroys superoxide anion radicals normally produced within the cells, was found in 2.2 heart slices^26^. We found heat shock proteins facilitate the correct folding of imported proteins such as Hspe1 or Hspd1, which were up-regulated too. It has been proven that heat shock proteins have many different cardioprotective functions, which include preventing apoptosis pathway activation, inhibiting proinflammatory cytokines, restoring redox balance, repairing ion channels, and interacting with nitric oxide-induced protection^27,28^.

In our proteomic analysis, only one protein-osteoglycin was statistically significant and present at more than a twofold concentration change in a comparison of 2.2 to C when 19 such proteins for TW–C in that comparison were found. In addition, osteoglycin was found to be an important regulator of left ventricular mass in rats, mice, and humans^29,30^. Moreover, it prevents the development of age-related diastolic dysfunction during pressure overload by reducing cardiac fibrosis and inflammation^29^.

## Limitations of the study

Proteomic composition and endogenous peptides may become excellent biomarkers of the heart slice condition in in vitro culture. On the other hand, it is crucial to keep in mind all the limitations of this scientific approach like that ultrathin heart slices are not easy to prepare, then peptides can be easily bound by various proteins. This event may cause differences in quantitative response in sample repetitions due to peptide retention on proteins during sample preparation. As a result, observed changes in the measured relative concentration of the particular peptide, may not reflect a real situation in the sample. The presence of the protein fragments and changes in the peptide concentrations could be used to describe the condition and the physiological/pathological state of the myocardium. The results obtained from quantitative proteomic analysis of different approaches to heart slices could be used as biomarkers.

## Conclusion

The proteomic pattern of the heart slices under biomimetic electromechanical stimulation with a relative quantification technique SWATH-MS, allowing label-free quantification of virtually any number of proteins in one analysis, has never been studied before. This study demonstrated a proteomic pattern shift between rat myocardial slices that were stretched and fresh myocardial slices cultured without electromechanical stimulation. The most prominent changes were observed in the proteins related to mitochondrial respiration, energy metabolism, and muscle contraction. The results give absolute concentrations for 399 proteins spanning more than 4 orders of magnitude, which gives a good resource to study.

## Supplementary Information

Supplementary Information 1.

Supplementary Information 2.

Supplementary Information 3.

## Data Availability

The datasets generated and analyzed during the current study are available in the ProteomeXchange Consortium via the PRIDE partner repository, with the dataset identifier PXD034891^31^.
