# Supplementary material for: Label-free quantitative SWATH-MS proteomic analysis of adult myocardial slices in vitro after biomimetic electromechanical stimulation
Source: Sci Rep. 2022 Oct 3;12:16533. doi: 10.1038/s41598-022-20494-z (PMC9529937; doi:10.1038/s41598-022-20494-z)
Supplement: Supplementary file 1 — Supplementary Information 1. [file 41598_2022_20494_MOESM1_ESM.pdf]

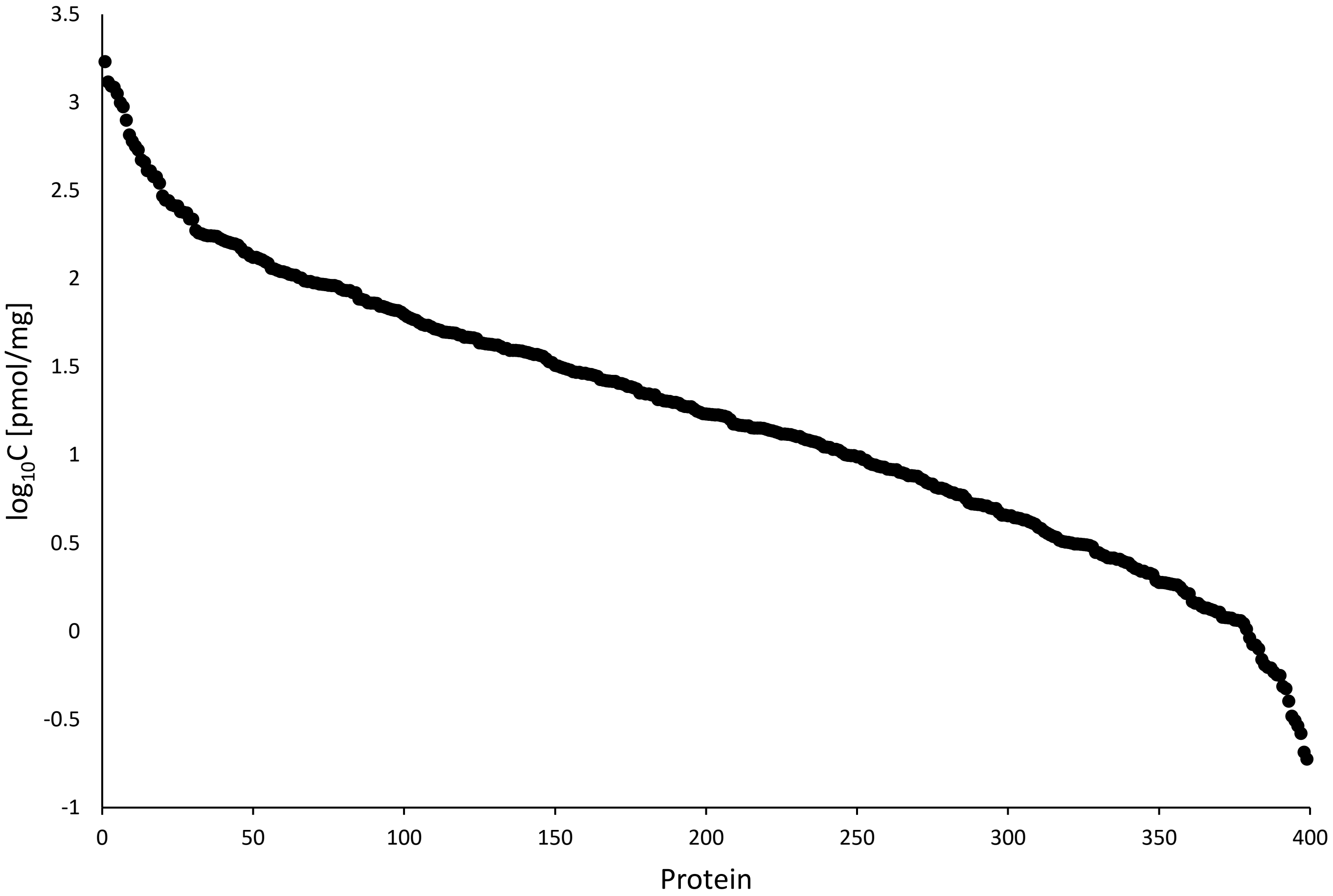

Figure S1. Concentration range of protein quantification. Median concentration values (log_{10} [pmol/mg]) in the control group are shown for all quantified proteins in the descending order.

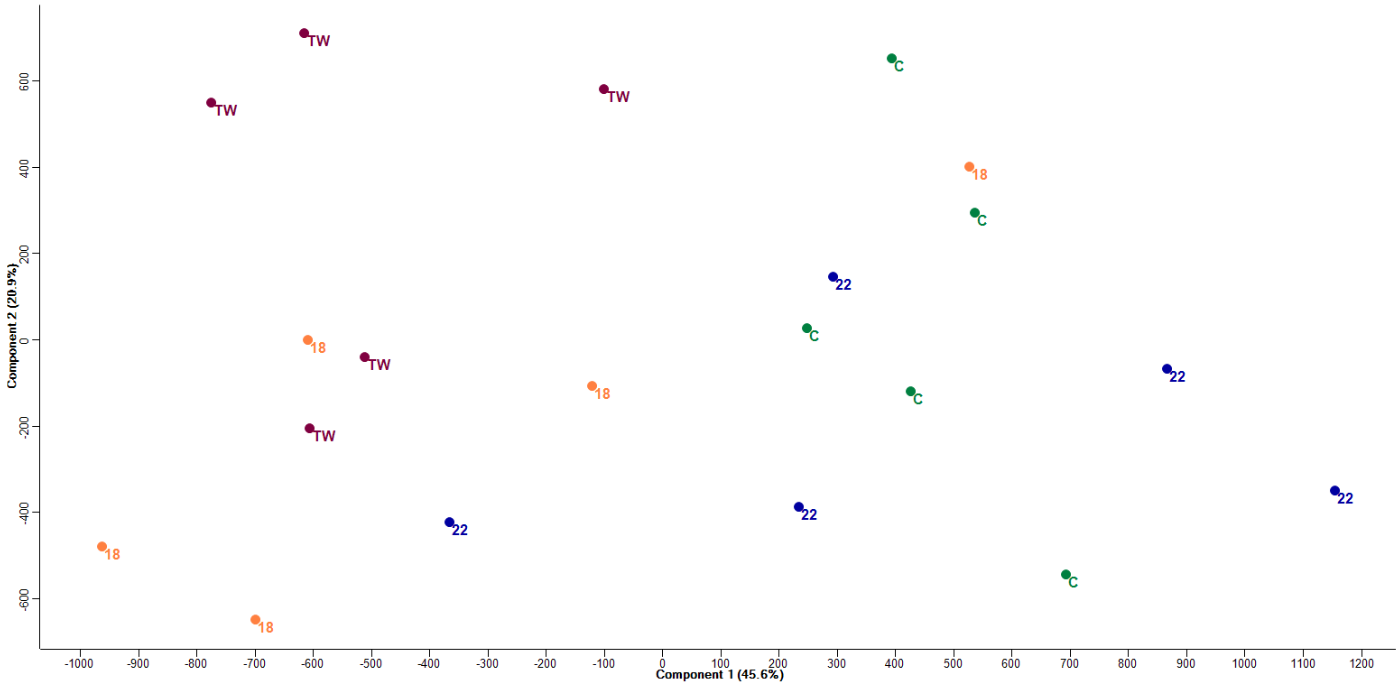

Figure S2. Graphical representation of the principal component analysis results for all samples analyzed in this study with the group designation: control (C) – green, 1.8 (18) – orange, 2.2 (22) – blue, TW – purple.

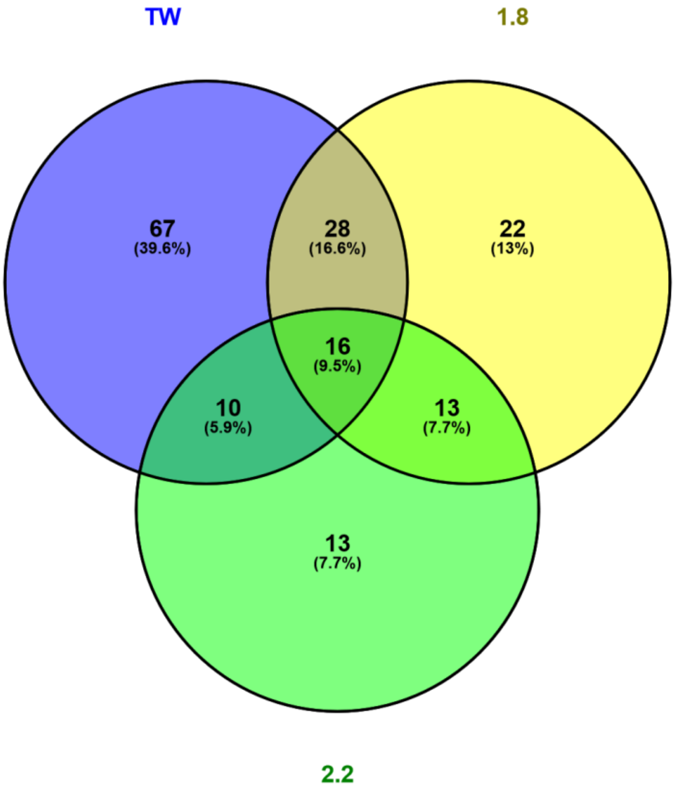

Figure S3. Vennn plot presenting sets of proteins with statistically significant changes in concentration in comparison to the control group.
