# Supplementary material for: Label-free quantitative SWATH-MS proteomic analysis of adult myocardial slices in vitro after biomimetic electromechanical stimulation
Source: Sci Rep. 2022 Oct 3;12:16533. doi: 10.1038/s41598-022-20494-z (PMC9529937; doi:10.1038/s41598-022-20494-z)
Supplement: Supplementary file 3 — Supplementary Information 3. [file 41598_2022_20494_MOESM3_ESM.docx]

Table SM2. List of proteins with the highest concentration changes (fold change>2 in at least one comparison) at p-value<0.05.

|  |  | **Median concentration [pmol/mg]** | | | | **Fold change** | | | **p-value** | | |
| --- | --- | --- | --- | --- | --- | --- | --- | --- | --- | --- | --- |
| **Uniprot ID** | **Protein name** | **C** | **TW** | **1_8** | **2_2** | **TW to C** | **18 to C** | **22 to C** | **TW to C** | **18 to C** | **22 to C** |
| P12346 | Serotransferrin | 4.52 | 12.43 | 2.59 | 2.21 | **2.75** | 0.57 | 0.49 | 7.36E-03 | 2.40E-01 | 1.07E-01 |
| G3V913 | Heat shock 27kDa protein 1 | 17.44 | 35.51 | 25.64 | 21.75 | **2.04** | 1.47 | 1.25 | 3.27E-04 | 3.17E-02 | 1.26E-01 |
| O35567 | Bifunctional purine biosynthesis protein PURH | 0.92 | 1.84 | 1.38 | 1.24 | **2.01** | 1.51 | 1.36 | 3.30E-02 | 9.97E-02 | 5.97E-01 |
| P19511 | ATP synthase F(0) complex subunit B1, mitochondrial | 92.60 | 45.73 | 49.19 | 86.75 | **0.49** | 0.53 | 0.94 | 5.07E-03 | 1.56E-02 | 5.67E-01 |
| P07483 | Fatty acid-binding protein, heart | 348.27 | 169.12 | 295.67 | 434.51 | **0.49** | 0.85 | 1.25 | 1.77E-02 | 9.23E-01 | 3.43E-01 |
| Q6IE67 | Proteasome subunit alpha type | 3.17 | 1.43 | 1.53 | 2.12 | **0.45** | 0.48 | 0.67 | 4.53E-02 | 1.09E-01 | 5.75E-01 |
| F1LX07 | Solute carrier family 25 member 12 | 4.41 | 1.99 | 2.33 | 3.28 | **0.45** | 0.53 | 0.74 | 4.57E-03 | 6.87E-02 | 3.49E-01 |
| B0BMY8 | Histone H3 | 31.89 | 14.22 | 16.09 | 17.11 | **0.45** | 0.50 | 0.54 | 4.03E-03 | 1.69E-03 | 2.36E-02 |
| F1LZW6 | Solute carrier family 25 member 13 | 12.14 | 5.14 | 6.40 | 6.48 | **0.42** | 0.53 | 0.53 | 3.63E-02 | 2.42E-01 | 2.03E-01 |
| G3V8C4 | Chloride intracellular channel protein | 4.72 | 1.98 | 4.16 | 4.29 | **0.42** | 0.88 | 0.91 | 5.35E-03 | 9.89E-01 | 4.25E-01 |
| A9UMV7 | RCG29512 | 42.41 | 17.35 | 21.76 | 54.51 | **0.41** | 0.51 | 1.29 | 6.78E-03 | 3.72E-02 | 9.17E-01 |
| Q5BK63 | NADH dehydrogenase [ubiquinone] 1 alpha subcomplex subunit 9, mitochondrial | 49.35 | 20.09 | 25.28 | 42.76 | **0.41** | 0.51 | 0.87 | 1.19E-03 | 2.31E-02 | 3.87E-01 |
| Q5FVT5 | Pyruvate dehydrogenase kinase, isozyme 1 | 2.19 | 0.87 | 1.28 | 1.56 | **0.40** | 0.59 | 0.71 | 3.28E-02 | 6.91E-01 | 7.69E-01 |
| F1LRT9 | Cytoplasmic dynein 1 heavy chain 1 | 0.19 | 0.07 | 0.10 | 0.15 | **0.38** | 0.53 | 0.80 | 1.21E-02 | 1.93E-01 | 8.35E-01 |
| O70253 | Muscle carnitine palmitoyltransferase I | 4.53 | 1.40 | 3.67 | 3.24 | **0.31** | 0.81 | 0.72 | 1.39E-03 | 1.42E-01 | 1.94E-01 |
| P84817 | Mitochondrial fission 1 protein | 13.04 | 6.32 | 5.61 | 11.72 | **0.48** | **0.43** | 0.90 | 8.93E-03 | 3.28E-03 | 5.28E-01 |
| P62142 | Serine/threonine-protein phosphatase PP1-beta catalytic subunit | 8.23 | 3.17 | 3.97 | 5.43 | **0.39** | **0.48** | 0.66 | 3.61E-04 | 1.49E-02 | 2.50E-02 |
| Q4V8H8 | EH domain-containing protein 2 | 2.78 | 1.01 | 1.22 | 1.88 | **0.36** | **0.44** | 0.67 | 6.31E-03 | 9.65E-03 | 4.78E-01 |
| P53534 | Glycogen phosphorylase, brain form (Fragment) | 13.36 | 3.67 | 6.38 | 7.48 | **0.27** | **0.48** | 0.56 | 5.05E-05 | 3.84E-03 | 4.50E-02 |
| P45592 | Cofilin-1 | 12.22 | 19.59 | 25.44 | 14.36 | 1.60 | **2.08** | 1.18 | 2.14E-02 | 2.79E-03 | 2.79E-01 |
| B2RYS0 | Cox7a2 protein | 10.04 | 9.68 | 22.36 | 10.84 | 0.96 | **2.23** | 1.08 | 6.01E-01 | 1.94E-02 | 5.97E-01 |
| A0A096MK30 | Moesin | 2.80 | 1.60 | 1.35 | 1.92 | 0.57 | **0.48** | 0.69 | 1.60E-04 | 3.24E-03 | 9.88E-02 |
| Q08163 | Adenylyl cyclase-associated protein 1 | 5.25 | 3.31 | 2.41 | 3.48 | 0.63 | **0.46** | 0.66 | 1.22E-01 | 3.11E-02 | 3.93E-01 |
| P11507-2 | Isoform 2 of Sarcoplasmic/endoplasmic reticulum calcium ATPase 2 | 56.35 | 48.04 | 24.37 | 45.34 | 0.85 | **0.43** | 0.80 | 5.83E-01 | 4.01E-02 | 1.97E-01 |
| F1LZC5 | NADH:ubiquinone oxidoreductase subunit A13 | 536.87 | 310.97 | 169.90 | 406.73 | 0.58 | **0.32** | 0.76 | 8.66E-03 | 1.69E-02 | 6.96E-01 |
| A0A0G2K926 | Alpha-1-inhibitor III | 1.38 | 2.21 | 0.34 | 0.51 | 1.60 | **0.25** | 0.37 | 1.32E-01 | 2.95E-02 | 7.77E-02 |
| D3ZVB7 | Osteoglycin | 8.51 | 7.32 | 2.83 | 3.73 | 0.86 | **0.33** | **0.44** | 9.29E-01 | 3.91E-04 | 1.47E-03 |
